# Supplementary material for: Expanded quantum vortex liquid regimes in the electron nematic superconductors FeSe1−xSx and FeSe1−xTex
Source: Nat Commun. 2023 Jul 12;14:4150. doi: 10.1038/s41467-023-39730-9 (PMC10338513; doi:10.1038/s41467-023-39730-9)
Supplement: Supplementary file 1 — Supplementary Information [file 41467_2023_39730_MOESM1_ESM.pdf]

Supplementary Information for

Expanded quantum vortex liquid regimes in the electron nematic superconductors $FeSe_{1-x}S_x$ and $FeSe_{1-x}Te_x$

M. Čulo, S. Licciardello *et al*.

Supplementary Note 1

Dependence of H_{irr} on the applied current in $FeSe_{1-x}S_x$. As argued in the main text, in order to determine the *H*-*T* phase diagram of a type-II superconductor by dc resistivity measurements, one has to fulfill the condition that the irreversibility line is sufficiently close to the melting line, i.e. $H_{irr}(T) \approx H_m(T)$. Such a condition can be experimentally realized by using low current excitations, i.e. by working in the limit $j \rightarrow 0$, so that the Lorentz force is always smaller than the pinning force. In other words, the onset of a finite resistivity is related to a vortex solid-liquid transition, not to the unpinning of the vortex solid.

In this work we used low current excitations in the range 0.5-1 mA, which is the minimum excitation with a good signal to noise ratio. Nevertheless, in order to show that at these current excitations the approximation $j \rightarrow 0$ holds, we checked for a possible dependence of H_{irr} on the applied current. This test was performed on the sample FSS25, for which the quantum vortex liquid (QVL) regime is the most pronounced.

The resulting magnetoresistance (MR) curves are shown in Supplementary Figure 1 at two different temperatures, T = 0.61 K and T = 1.8 K, and for current excitations in the range 30 μA – 1 mA. Here H_{irr} is defined as the field beyond which resistivity rises above the noise level at the zero-value. As we can see, though the MR curves slightly differ for different current excitations, a 30-fold decrease in excitation current leads to changes in H_{irr} within a small range 6-7 T at 0.61 K. Taking into account the average value of $H_{c2} \approx 20$ T, this implies that the ratio $H_{irr}(0)/H_{c2}(0)$, plotted in Figure 3H of the main article, changes only within the range 0.3 - 0.35. The main conclusion of the present study therefore stays unaffected, supporting the approximation of the zero current limit $j \rightarrow 0$.

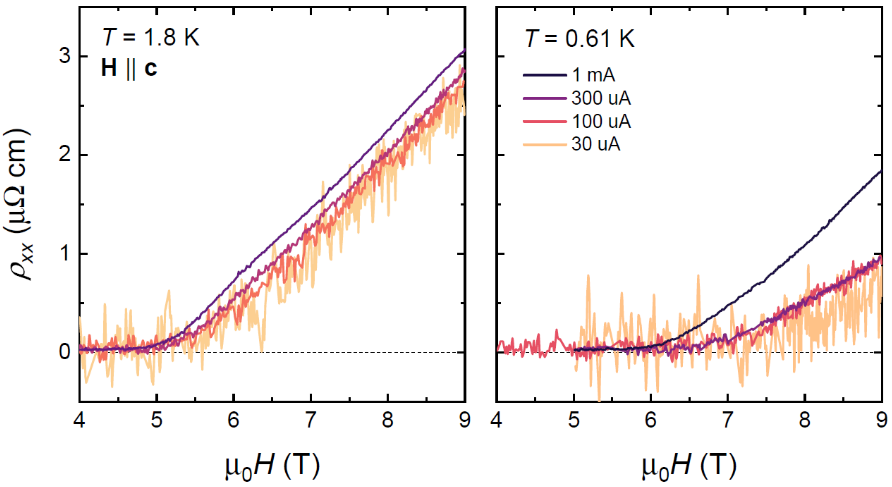

Supplementary Figure 1. Dependence of H_{irr} on the applied current for FSS25 at T = 1.8 K (left panel) and T = 0.61 K (right panel) in the transverse field orientation **H**||*c*. Here H_{irr} is defined as the field beyond which resistivity rises above the noise level at the zero-value.

Supplementary Note 2

Determination of H_{c2} from the transverse MR in $FeSe_{1-x}S_x$. As shown previously (1, 2), the normal state MR of $FeSe_{1-x}S_x$ in the transverse field orientation **H**||*c* is large and positive and exhibits a complex behaviour that makes estimating a reliable value for $H_{c2}(T)$ more challenging than for **H**||*ab*. Adopting a procedure that exploits the universal behaviour of the MR derivative $d\rho_{xx}/d(\mu_0 H)$ (1), however, enables $H_{c2}(T)$ to be determined with reasonable accuracy. The behaviour of $d\rho_{xx}/d(\mu_0 H)$ in $FeSe_{1-x}S_x$ for **H**||*c* in the temperature interval $0.3\ K \leq T \leq 15\ K$ is summarized in Supplementary Figure 2 for all measured samples. In each panel, all curves are normalized to their value at the highest field (38 T). The black dashed lines in Supplementary Figure 2 represent the normalized MR derivatives at a temperature that is considered to be above the SC transition where no signatures of SC fluctuations are visible and the derivatives reflect the behaviour of the transverse MR in the normal state. Except for pure FeSe, above a certain *T*-dependent field scale, all normalized $d\rho_{xx}/d(\mu_0 H)$ curves are found to collapse onto a single line (on top of which quantum oscillations can be seen). This overlapping of each successive curve implies that below around 15 K, the form of $d\rho_{xx}/d(\mu_0 H)$ becomes essentially independent of temperature in the normal state beyond $H_{c2}(T)$. The high-field behaviour itself can be described by a straight line with a finite intercept indicating that $\rho_{xx}(H) \propto H + H^2$ at high fields (1). In these plots, the black dashed lines serve as base lines for the determination of $H_{c2}(T)$, the latter being determined from the field scale above which each $d\rho_{xx}/d(\mu_0 H)$ curve overlaps with the former, as indicated by the vertical arrows in Supplementary Figure 2. $H_{irr}(T)$ is determined in the same way as for the longitudinal field orientation, from the onset field beyond which the MR derivative steeply rises above the noise level at zero value. The only sample for which it is not possible to determine $H_{c2}(T)$ from this procedure is FSS00, whose transverse MR exhibits a *T*- and *H*-dependence that is too strong and as a result, there is no overlap between the normalized $d\rho_{xx}/d(\mu_0 H)$ curves. Consequently, its $H_{irr}(0)/H_{c2}(0)$ ratio is not plotted in Figure 3 of the main manuscript.

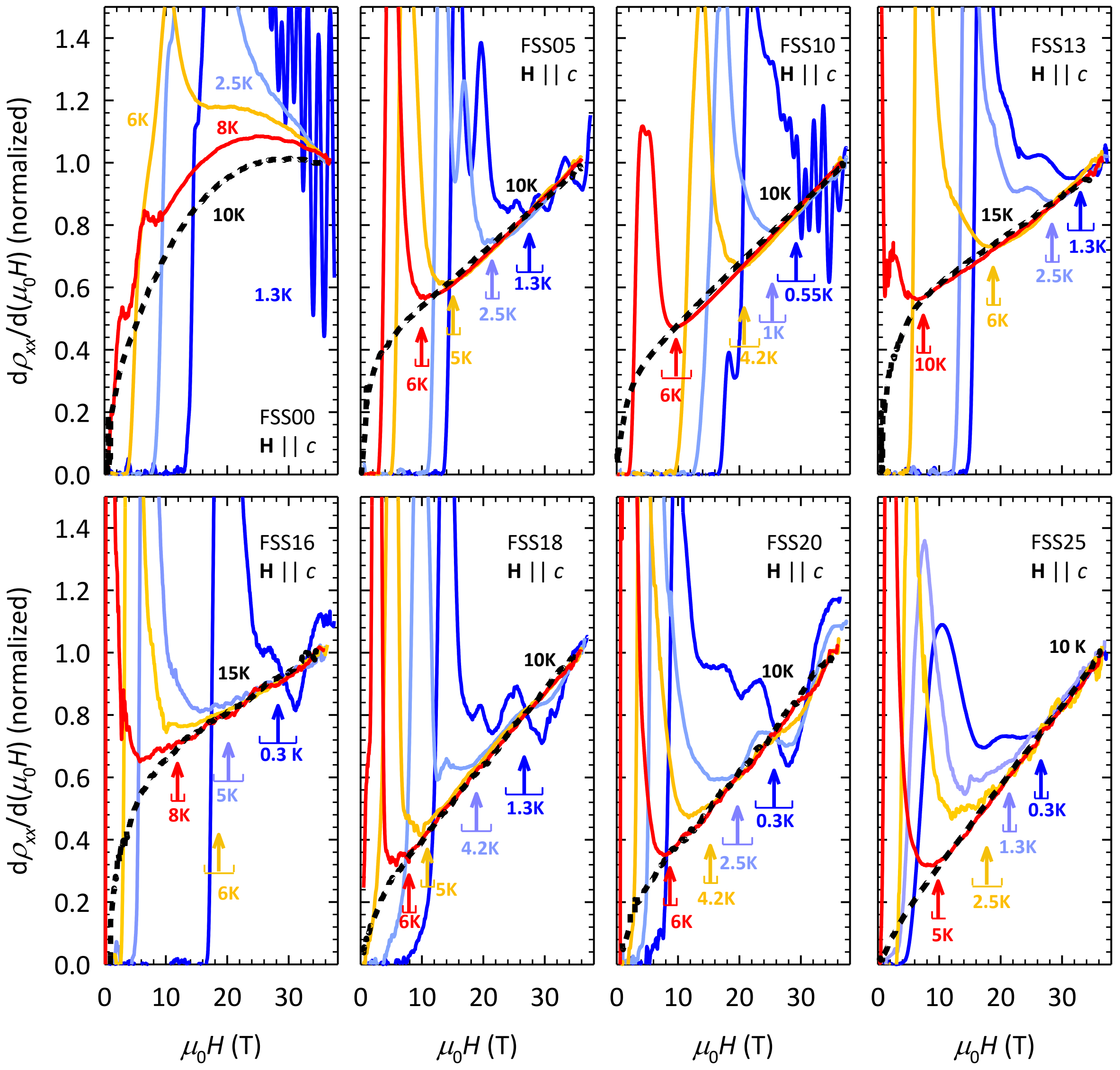

Supplementary Figure 2. Determination of H_{irr} and H_{c2} in $FeSe_{1-x}S_x$ in the transverse field orientation **H**||*c*. Shown are the MR derivatives $d\rho_{xx}/d(\mu_0 H)$ normalized to the value at the maximum field 38 T for: FSS00, FSS05, FSS10, FSS13, FSS16, FSS18, FSS20 and FSS25. Black dashed curves represent the MR derivatives obtained at temperatures above the SC transition where no signatures of SC fluctuations are visible. Red, yellow, light blue and blue lines, in that order, refer to the MR derivatives below the SC transition at selected decreasing temperatures. H_{irr} is defined as the onset field for finite resistivity and determined by the field value at which the MR derivative rises above the noise level. H_{c2} is determined by the field value at which the MR derivatives start to overlap with the dashed curves, as indicated by vertical arrows. The error bars for H_{c2} are estimated from the noise level and are indicated by the horizontal bars below each arrow. The curves at the lowest temperatures show the appearance of quantum oscillations at the highest fields which introduces additional uncertainty in our estimate for H_{c2}. The absence of such overlap in FSS00 prevents a reliable determination of H_{c2} for pure FeSe (see text).

Supplementary Note 3

Determination of critical fields from the Hall effect in $FeSe_{1-x}S_x$. Due to its multiband and dual character (i.e., containing both conventional and 'strange' metallic components (3)), the normal state Hall resistivity ρ_{yx} of $FeSe_{1-x}S_x$ exhibits highly non-linear and strongly T-dependent behaviour. We have therefore employed a similar procedure to that used for the transverse MR and exploited the weaker T-dependence of the (normalized) derivatives, as illustrated in Supplementary Fig. 3. As in Supplementary Fig. 2, black dashed lines represent the $d\rho_{yx}/d(\mu_0 H)$ curves obtained at a temperature where no signatures of SC fluctuations are present. Plotted in this way, the normalized $d\rho_{yx}/d(\mu_0 H)$ curves tend to overlap with the dashed lines in the high field region, at which point we assume that the sample has reached the field-induced normal state. $H_{c2}(T)$ is then deduced from the onset field value at which this overlap occurs, as indicated by the vertical arrows in Supplementary Fig. 3. As with the transverse MR study, the only exception is FSS00 (not shown) where the lack of overlap with the dashed curve, especially at low T, prevents a reliable determination of $H_{c2}(T)$. For all samples, $H_{irr}(T)$ is again determined by the onset field beyond which the (Hall) derivative rises above the noise level.

The resultant $H_{irr}(0)/H_{c2}(0)$ ratios determined from the Hall measurements are compared in Supplementary Figure 4 with the corresponding values extracted from the transverse MR study. As we can see, there is good agreement between the two data sets across the $FeSe_{1-x}S_x$ series, giving us added confidence in our determination of $H_{irr}(0)/H_{c2}(0)$ for **H**||c.

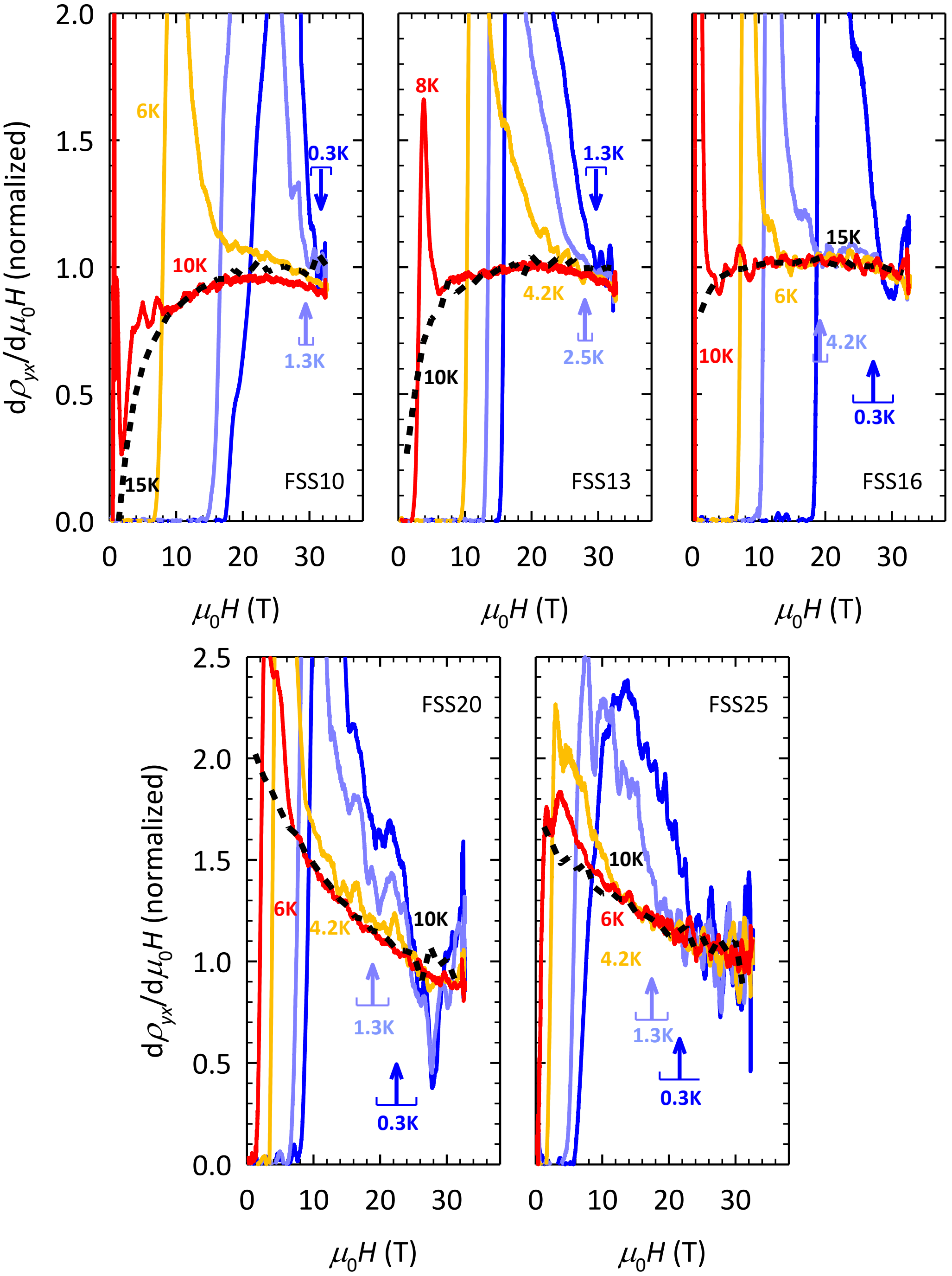

Supplementary Figure 3. Determination of $H_{irr}(T)$ and $H_{c2}(T)$ in $FeSe_{1-x}S_x$ from Hall effect measurements. Shown are the Hall derivatives $d\rho_{yx}/d(\mu_0 H)$ normalized to the value at the maximum field 33 T for FSS10, FSS13, FSS16, FSS20 and FSS25. Black dashed curves are the Hall derivatives obtained at a temperature above the SC transition where no signatures of SC fluctuations are visible. Red, yellow, light blue and blue lines, in that order, refer to the Hall derivatives below the SC transition at selected decreasing temperatures. H_{irr} is defined as the onset field for finite resistivity and determined by the field value at which the Hall derivative rises above the noise level. H_{c2} is determined by the field value at which the Hall derivatives overlap with the dashed curves, as indicated at the lowest temperatures by the vertical arrows. The error bars for H_{c2} are estimated from the noise level and are indicated by the horizontal bars below each arrow. The curves at the lowest temperatures show the appearance of quantum oscillations at the highest fields which introduces additional uncertainty in our estimate for H_{c2}. As with the transverse MR, the strong H- and T-dependence of $d\rho_{yx}/d(\mu_0 H)$ in FSS00 precludes a reliable determination of H_{c2} for pure FeSe.

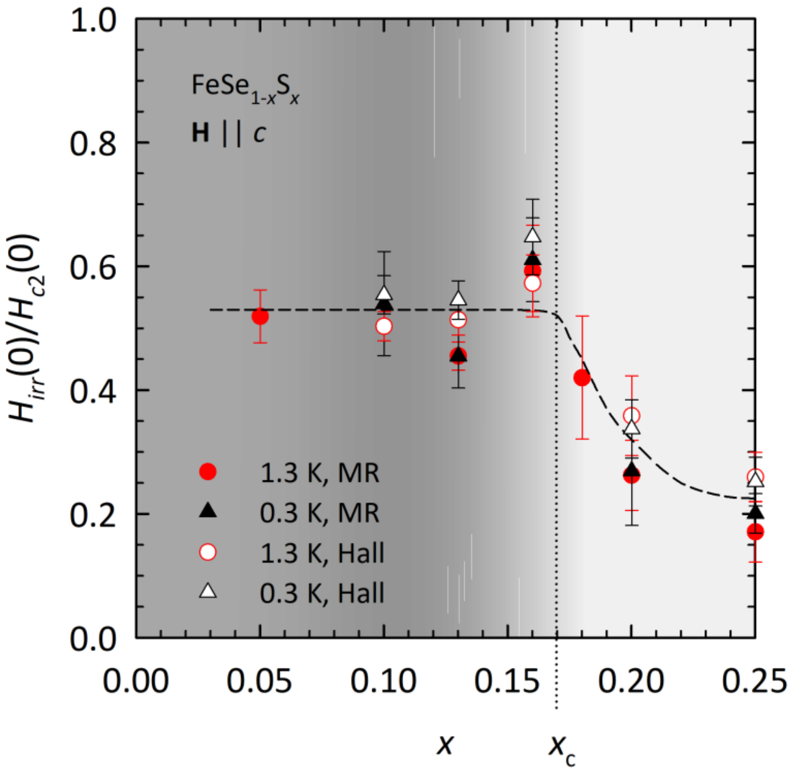

Supplementary Figure 4. Comparison of $H_{irr}(0)/H_{c2}(0)$ extracted from MR (full symbols) and Hall resistivity (empty symbols) measurements at T = 0.3 K (circles) and 1.3 K (triangles) with **H**||c. As we can see, there is good agreement in the values of $H_{irr}(0)/H_{c2}(0)$ obtained by the two methods across the $FeSe_{1-x}S_x$ series. The error bars for $H_{irr}(0)/H_{c2}(0)$ are composite errors obtained from the errors in H_{irr} and H_{c2}. (The error bars for H_{c2} are taken from Supplementary Figures 2 and 3, and for H_{irr} are estimated from the threshold noise.)

Supplementary Note 4

Alternative procedures for determining critical fields from the MR in $FeSe_{1-x}S_x$. The critical fields $H_{irr}(T)$ and $H_{c2}(T)$ shown in Figure 3 of the main article were determined from the derivatives of the longitudinal resistivity $d\rho/d(\mu_0 H)$. Here we present two alternative procedures for obtaining H_{c2} directly from the measured resistivity ρ_{xx} that remove, to a greater or lesser extent, the enhancement of H_{c2} due to SC order parameter fluctuations.

In the first procedure, H_{c2} is deduced from the field at which $\rho_{xx}(H)$ reaches 95% of ρ_n, where ρ_n corresponds to the normal state resistivity at the H_{c2} value originally obtained from the derivative plots. This simple procedure is illustrated by the horizontal dotted lines in Supplementary Figure 5 for two representative concentrations: FSS13, to the left, and FSS25, to the right of the nematic QCP, for both the longitudinal and transverse field orientations. Clearly, for a sample with a broad SC fluctuation regime this procedure will reduce H_{c2} by a larger extent that on a sample with a narrow fluctuation regime.

In the second procedure (similar to that reported recently in Ref. (4) and also demonstrated in Supplementary Figure 5 for the same two data sets), we first make two straight-line fits to the MR data, one in the mixed (vortex) state, and the other in the normal state. The fits in the mixed state were performed in the vicinity of the maximum in the field derivatives $d\rho/d(\mu_0 H)$. The normal-state fits were defined depending on the field orientation. In the longitudinal field orientation, where the normal-state MR is either negligible or small and linear (5), the fits were performed at the highest measured field strengths (see panels A and B of Supplementary Figure 5). For **H**||*c*, on the other hand, the stronger field dependence of the MR confined the linear fits to a narrow field range just beyond the maxima in the field derivatives (see panels E and F of Supplementary Figure 5). H_{c2} is then deduced from the field at which the two straight-line fits intersect.

Comparison of panels E and F in Supplementary Fig. 5 with the corresponding 1.3 K derivative plots shown in Supplementary Fig. 2 reveals that at $H = H_{c2}^B$ – the value for H_{c2} deduced by method B – the slopes of the MR curves are approximately twice their expected value at H_{c2}^A (i.e. in the absence of SC fluctuations), even though this change is hard to discern fro the raw $\rho(H)$ curves. Inevitably then, the H_{c2} values determined from $\rho(H)$ are smaller than the ones obtained from $d\rho/d(\mu_0 H)$. Nevertheless, despite the differences in the absolute values of $H_{irr}(0)$ and $H_{c2}(0)$, the overall trend in the *x*-dependence of their ratio remains qualitatively the same. (Throughout this analysis, $H_{irr}(T)$ is always defined as the field value above which ρ_{xx} rises above a threshold set by the noise level in the measurement.) If we include also the analysis based on the Hall effect data discussed in Supplementary note 3, the quantitative conclusions summarized in Figure 3 of the main article using the derivative analysis can now be broadened out to encompass all four procedures. For **H**||*ab*, $H_{irr}(0)/H_{c2}(0) \approx 0.8 - 0.95$ for $x \leq x_c$ implying that the QVL regime is narrow or non-existent. Beyond x_c, $H_{irr}(0)/H_{c2}(0)$ drops to a value 0.4 - 0.6, depending on the criterion used to determine $H_{c2}(0)$, indicating a marked broadening of the QVL regime outside of the nematic phase. In the transverse configuration, $H_{irr}(0)/H_{c2}(0)$ is again roughly constant for $x \leq x_c$, but always with a value that is lower than for **H**||*ab*, irrespective of the procedure used, while beyond x_c, the ratio drops even further, indicating the presence of a robust QVL regime across the nematic QCP.

Although probably too restrictive, we find the method based on resistivity derivative to be superior to the ones based on raw resistivity data. First of all, in contrast to the resistivity method, the method based on derivative does not assume any specific form of MR in the normal state and is therefore free of approximations, which is especially important for the transverse field orientation. Secondly, the derivative method does not rely on the fitting procedure which is sometimes limited to a narrow field range (see Supplementary Fig. 5A) which can have a significant impact on the estimate of H_{c2}. Finally, the method based on the derivatives partially averages out the noise in the raw resistivity data making it easier to detect the change in behaviour. The chief disadvantage of the derivative method is that it will inevitably lead to an overestimate of H_{c2} by virtue of the fact that it is the field scale beyond which all signatures of SC fluctuations are lost.

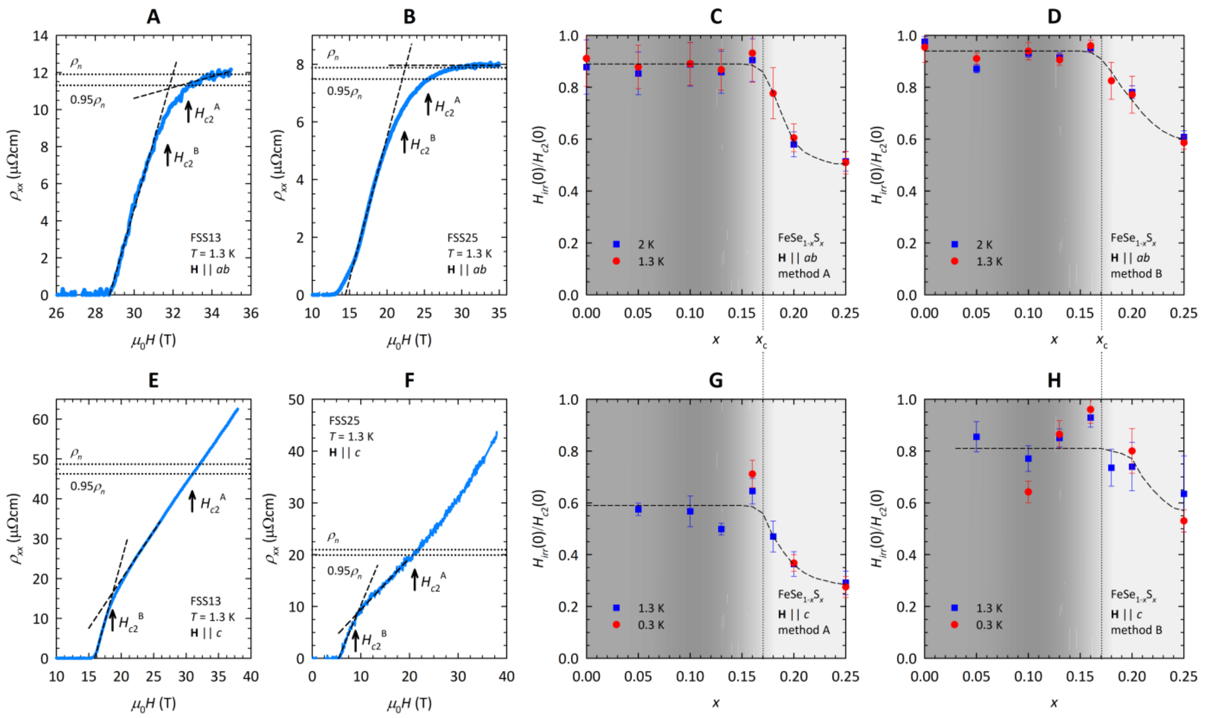

Supplementary Figure 5. Alternative estimates of $H_{c2}(0)$ determined directly from the measured resistivity $\rho(H)$. Panels **A**, **B**, **E** and **F** show $\rho(H)$ at T = 1.3 K for (**A**) FSS13 and (**B**) FSS25 with **H**||*ab* and for (**E**) FSS13 and (**F**) FSS25 with **H**||*c*, respectively. H_{irr} is defined as the field value above which ρ_{xx} rises above a threshold – set by the noise level in the zero-resistivity state. H_{c2} is determined in two different ways. In method **A**, the upper critical field H_{c2}^A is defined as the field at which ρ_{xx} reaches 95% of the normal state resistivity ρ_n, where ρ_n corresponds to the normal state resistivity at the H_{c2} value originally obtained from the derivative plots, indicated by dotted lines. In method **B**, the upper critical field H_{c2}^B is determined from the intersection of straight-line fits to the mixed (vortex) state and normal state resistivity (see text) indicated by the black dashed lines. The extracted $H_{irr}(0)/H_{c2}(0)$ ratios for method **A** are shown in panels **C** (**H**||*ab*) and **G** (**H**||*c*), and for method **B** in panels **D** (**H**||*ab*) and **H** (**H**||*c*). The error bars for $H_{irr}(0)/H_{c2}(0)$ are composite errors obtained from the errors in H_{irr} and H_{c2}. (The error bars for H_{irr} are estimated from the threshold noise and for H_{c2} from the corresponding fitting procedures.)

Supplementary Note 5

Determination of H_{c2} from the transverse MR in $FeSe_{1-x}Te_x$. H_{c2} in $FeSe_{1-x}Te_x$ for $\mathbf{H}||c$ is determined in the same way as for the sister family $FeSe_{1-x}S_x$ shown in Supplementary Figure 2. The procedure is illustrated in Supplementary Figure 6 for three representative Te-substitutions: $x = 0.11$, which is deep inside the nematic phase, $x = 0.30$, which is close to the dip in $H_{irr}(0)/H_{c2}(0)$ where T_c has a minimum and the QVL regime is most pronounced (see Figure 4 in the main article), and $x = 0.80$, which lies outside of the nematic phase. The black dashed lines represent the MR derivatives at a temperature close to the SC transition which should reflect the behaviour of the transverse MR in the normal state. Note the SC fluctuations at the lowest fields indicating that the system has not yet completely reached the normal state. These fluctuations, however, do not influence the high-field behaviour, where the overlaps happen, and therefore do not influence on the determination of H_{c2}. Since the MR derivatives in $FeSe_{1-x}Te_x$ become almost negligible for $x > 0.5$, only the curves in the nematic phase are normalized to their value at the highest field (35 or 55 T). The marked enhancement of the QVL regime for $x = 0.30$ relative to $x = 0.80$ is clear from comparison of the last two panels.

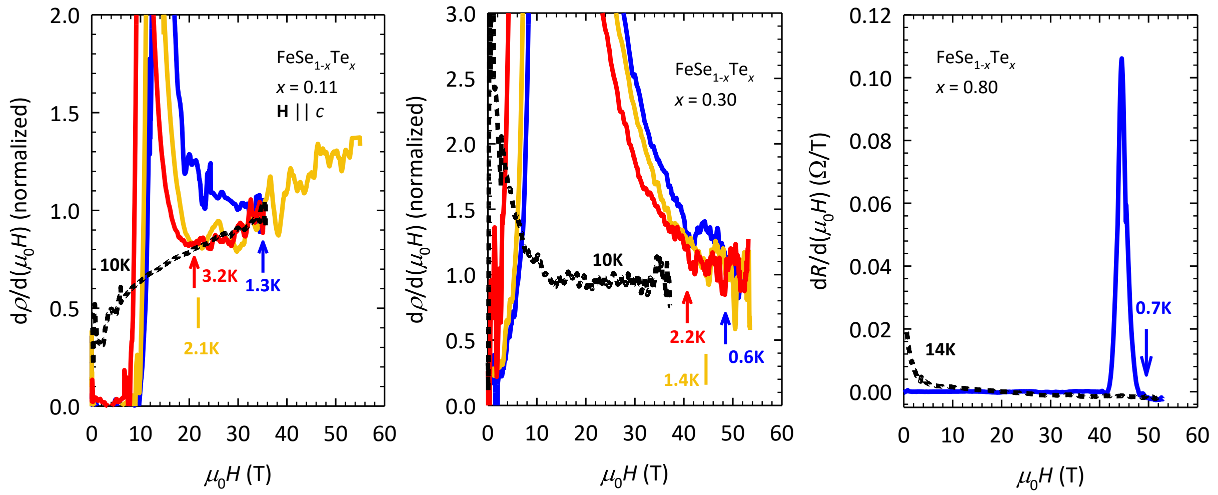

Supplementary Figure 6. Determination of H_{irr} and H_{c2} in $FeSe_{1-x}Te_x$ in the transverse field orientation $\mathbf{H}||c$. Shown are the MR derivatives $dR/d(\mu_0 H)$ for three representative Te-substitutions: $x = 0.11$, $x = 0.30$, and $x = 0.80$. The MR derivatives are normalized to their value at the highest field (35 or 55 T) except for $x = 0.80$, where the MR derivative is close to zero at high fields. Black dashed curves represent the MR derivatives obtained at temperatures close to the SC transition where signatures of SC fluctuations are visible only at the lowest fields. Red, yellow, and blue lines, in that order, refer to the MR derivatives below the SC transition at selected decreasing temperatures. H_{irr} is defined as the onset field for finite resistivity and determined by the field value at which the MR derivative rises above the noise level. H_{c2} is determined by the field value at which the MR derivatives start to overlap with the dashed curves, as indicated by vertical arrows. The error bars for H_{irr} and H_{c2} are estimated from the noise level.

Supplementary Note 6

The BCS-BEC crossover in $FeSe_{1-x}S_x$. As mentioned in the main text, there is now growing evidence that $FeSe_{1-x}S_x$ lies close to a BCS-BEC crossover (6–8), an intermediate regime between the weak-coupling BCS limit, with strongly overlapping Cooper pairs (9), and the strong coupling BEC limit (10). One criterion that helps to establish whether a superconductor is located near the BCS-BEC crossover is the ratio between its superconducting gap Δ and its Fermi energy ε_F, which at the crossover takes the value $\Delta/\varepsilon_F \approx 1$. The vast majority of known superconductors are located deep in the BCS limit Δ/ε_F << 1. Based on quantum oscillations (11, 12), ARPES (12), STM (6, 13), London penetration depth (6) and thermopower (14) measurements, ε_F in FeSe is of the order of 10 meV, comparable to the reported superconducting gap of around 2-3 meV (13, 15, 16) which gives $\Delta/\varepsilon_F \approx 0.2 - 0.3$ and indicates that FeSe is closer to the BEC regime than most other superconductors (8).

In order to determine this ratio for the other members of the series, we have estimated the Fermi energy ε_F of $FeSe_{1-x}S_x$ as a function of x. This can be calculated by assuming parabolic pockets $\varepsilon_F = \hbar^2 k_F^2/(2m^*)$, where $\hbar$ is reduced Planck constant, k_F is the Fermi wave vector and m^* is the quasiparticle effective mass. k_F can be extracted from the frequencies F_i of quantum oscillations (17) using the Onsager relation $F_i = k_{Fi}^2/(2e)$, where i refers to one of the multiple frequencies and e is elementary charge. Note, however, that only the δ frequency has been detected in all samples up to x = 0.20 (17). The averaged frequencies F_{av} for both pockets are taken from Ref. (5) under the assumption that the compensation condition is always preserved. The F_{av} values for x = 0.25 are determined from the extrapolation of the data shown in Fig. 9 of Ref. (5). The variation in $m^*(x)$ is deduced from the coefficient of T^2 resistivity by the procedure explained in detail in Ref. (5).

The estimated Δ/ε_F ratios in $FeSe_{1-x}S_x$ thus obtained are listed in Supplementary Table 1. As we can see, the ratio becomes progressively smaller as x increases, reaching a value for x = 0.25 that is almost ten times smaller than for pure FeSe suggesting that with S substitution the system moves away from the crossover regime. Therefore, based on these estimates alone, one would conclude that proximity to the BCS-BEC crossover regime is not the primary origin of the expansion of the QVL phase in $FeSe_{1-x}S_x$. New experimental data (18, 19), however, show strong evidence for BEC-like superconductivity just beyond the nematic QCP. ARPES measurements (18), for example, claim to show strong evidence for BEC-like superconductivity beyond x_c, while the shape of the heat capacity jump at T_c is found to change markedly across x_c, exhibiting non-mean-field behaviour that is more reminiscent of a BEC transition (19). In light of these findings, we conclude that phenomena associated with the BCS-BEC crossover, while not the sole origin, are nevertheless playing some role in the realization of a broad QVL regime in $FeSe_{1-x}S_x$.

Supplementary Table 1. Estimates of the BCS-BEC crossover criterion in $FeSe_{1-x}S_x$. The SC energy gap Δ, Fermi energy ε_F and the ratio Δ/ε_F for a range of concentrations x studied here. The values of Δ are taken from Ref. (13).

x	0	0.05	0.10	0.13	0.16	0.18	0.20	0.25
Δ (meV)	2.55	2.35	2.4	2.3	1.95	1.6	1.35	1.3
ϵ_F (meV)	9.76	8.66	8.57	8.4	9.39	19.95	19.22	43.11
Δ/ϵ_F	0.26	0.27	0.28	0.27	0.21	0.08	0.07	0.03

Supplementary note 7

Characterization of the $FeSe_{1-x}S_x$ and $TeSe_{1-x}Te_x$ samples. The temperature of the nematic phase transition T_s in our $FeSe_{1-x}S_x$ samples is determined from the cusp in the resistivity derivative $\rho(T)$. The resultant evolution of T_s with x is shown in Figure 1(a) of Ref. (3), together with data points from a number of other studies (2,17,20-24). As can be seen there, our values for T_s(x) tend to lie above the average suggesting that the nominal values of x used here are higher than the actual x content (as determined by EDX). It is indeed possible that the quoted x values for our FSS10, FSS13, and FSS16 samples are an overestimate. For all other samples, however, both their resistivity and (low-field) Hall responses are identical to those with similar (but nonetheless EDX determined) x values reported elsewhere (17,20). More detailed discussion on this point can be found in Ref. (3).

Supplementary Figure 7 shows the x-evolution of residual resistivity of our $FeSe_{1-x}S_x$ samples. Here ρ_{res} was determined from the dc resistivity measurements at the maximum field of 35 T in the longitudinal field orientation **H**||*ab*, where MR is negligible for $x \geq 0.16$ and small and linear for $x < 0.16$. As we can see, ρ_{res} for FSS00 is only about 0.4 μΩcm, which to our knowledge is one of the lowest values reported for pure FeSe. For all the other samples $\rho_{res} \approx 10$ μΩcm, indicating that the sample quality does not vary significantly with S-substitution. The same conclusion can be drawn also from the width of the SC transition ΔT_c defined as a difference between the temperature at which the resistivity reaches 90 % of its normal state value and the temperature at which the resistivity rises above the noise level around zero-value. For all studied samples $\Delta T_c/T_c$ is in the range 0.15-0.3 with no obvious evolution with x. The residual resistivities for $FeSe_{1-x}Te_x$ samples are around $\rho_{res} \approx 0.3 \pm 0.1$ mΩcm (Fig. 1(a)-(d) in Ref. (25)). The corresponding transition temperatures, as deduced by magnetic susceptibility measruements, are shown in Fig. 1(a)-(d) of Ref. (26).

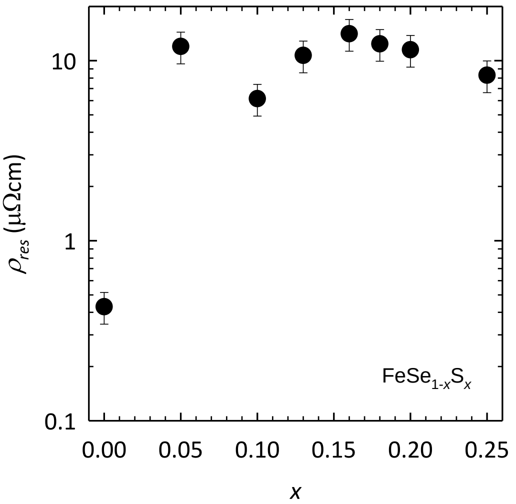

Supplementary Figure 7. The x-evolution of residual resistivity ρ_{res} of our $FeSe_{1-x}S_x$ samples determined from the dc resistivity measurements at the maximum field of 35 T in the longitudinal field orientation **H**||*ab*, where MR is negligible for $x \geq 0.16$ and small and linear for $x < 0.16$. The error bars for ρ_{res} are around 20 % due to the uncertainty in the sample geometry.

SI References

1. Licciardello, S. *et al*. Coexistence of orbital and quantum critical magnetoresistance in $FeSe_{1-x}S_x$. *Phys. Rev. Res.* **1**, 023011 (2019).
2. Bristow, M. *et al*. Anomalous high-magnetic field electronic state of the nematic superconductors $FeSe_{1-x}S_x$. *Phys. Rev. Res.* **2**, 013309 (2020).
3. Čulo, M. *et al*. Putative Hall response of the strange metal component in $FeSe_{1-x}S_x$. *Phys. Rev. Res.* 3, 023069 (2021).
4. Mukasa, K. *et al*. Enhanced superconducting pairing strength near a nonmagnetic nematic quantum critical point. *Phys. Rev. X* **13**, 011032 (2023).
5. Licciardello, S. *et al*. Electrical resistivity across a nematic quantum critical point. *Nature* **567**, 213–217 (2019).
6. Kasahara, S. *et al*., Field-induced superconducting phase of FeSe in the BCS-BEC cross-over. *Proc. Natl. Acad. Sci. (USA)* **111**, 16309–16313 (2014).
7. Hanaguri, T. *et al*. Quantum vortex core and missing pseudogap in the multiband BCS-BEC crossover superconductor FeSe. *Phys. Rev. Lett.* **122**, 077001 (2019).
8. Shibauchi, T., Hanaguri, T. & Matsuda, Y. Exotic superconducting states in FeSe-based materials. *J. Phys. Soc. Jpn.* **89**, 102002 (2020).
9. Kittel, C. *Introduction to Solid State Physics*. (Wiley, Hoboken, NJ), 8th edition, (2005).
10. Leggett, A. J. *Quantum Liquids*: *Bose condensation and Cooper pairing in condensed-matter systems,* Oxford Graduate Texts. (Oxford University Press, Oxford, New York), (2006).
11. Terashima, T. *et al*. Anomalous Fermi surface in FeSe seen by Shubnikov–de Haas oscillation measurements. *Phys. Rev. B* **90**, 144517 (2014).
12. Watson, M. D. *et al*. Emergence of the nematic electronic state in FeSe. *Phys. Rev. B* **91**, 155106 (2015).
13. Hanaguri, T. *et al*. Two distinct superconducting pairing states divided by the nematic end point in $FeSe_{1-x}S_x$. *Sci. Adv.* **4**, eaar6419 (2018).
14. Pourret, A. *et al*. Strong correlation and low carrier density in $Fe_{1+y}Te_{0.6}Se_{0.4}$ as seen from its thermoelectric response. *Phys. Rev. B* **83**, 020504 (2011).
15. Song, C. L. *et al*. Direct observation of nodes and twofold symmetry in FeSe superconductor. *Science* **332**, 1410–1413 (2011).
16. Sprau, P. O. *et al*. Discovery of orbital-selective Cooper pairing in FeSe. *Science* **357**, 75–80 (2017).
17. Coldea, A. I. *et al*. Evolution of the low-temperature Fermi surface of superconducting $FeSe_{1-x}S_x$ across a nematic phase transition. *npj Quantum Mater.* **4**, 2 (2019).
18. Hashimoto, T. *et al*. Bose-Einstein condensation superconductivity induced by disappearance of the nematic state. *Sci. Adv.* **6**, abb9052 (2020).
19. Mizukami,Y. *et al*. Thermodynamics of transition to BCS-BEC crossover superconductivity in $FeSe_{1-x}S_x$. *arXiV:2105.00739* (2021).
20. Huang, W. K. *et al*. Non-Fermi liquid transport in the vicinity of the nematic quantum critical point of superconducting $FeSe_{1-x}S_x$. *Phys. Rev. Res.* **2**, 033367 (2020).
21. Abdel-Hafiez, M. *et al*., Impurity scattering effects on the superconducting properties and the tetragonal-to-orthorhombic phase transition in FeSe. *Phys. Rev. B* **93**, 224508 (2016).
22. Wiecki, P. *et al*., Persistent correlation between superconductivity and antiferromagnetic fluctuations near a nematic quantum critical point in $FeSe_{1-x}S_x$. *Phys. Rev. B* **98**, 020507(R) (2018).
23. Hosoi, S. *et al*., Nematic quantum critical point without magnetism in $FeSe_{1-x}S_x$ superconductors. *Proc. Natl. Acad. Sci. (USA)* **113**, 8139 (2016).
24. Chareev, D. *et al*., Single crystal growth, transport and scanning tunneling microscopy and spectroscopy of $FeSe_{1-x}S_x$. *Cryst. Eng. Comm.* **20**, 2449 (2018).
25. Uezono, Y. *et al*., Onset Temperatures for Superconducting Fluctuations in Te-annealed $FeTe_{1-x}Se_x$ Single Crystals: Evidence for the BCS-BEC Crossover. *arXiv:2209.08238* (2022).
26. Otsuka, T. *et al*., Incoherent-coherent crossover and the pseudogap in Te-annealed superconducting $Fe_{1+y}Te_{1-x}Se_x$ revealed by magnetotransport measurements. *Phys. Rev. B* **99**, 184505 (2019).
